# Effect of metformin adjunct therapy on cardiometabolic parameters in Indian adolescents with type 1 diabetes: a randomized controlled trial

**DOI:** 10.3389/fcdhc.2024.1353279

**Published:** 2024-04-19

**Authors:** Shruti Mondkar, Sukeshini Khandagale, Nikhil Shah, Anuradha Khadilkar, Chirantap Oza, Shital Bhor, Ketan Gondhalekar, Aneeta Wagle, Neha Kajale, Vaman Khadilkar

**Affiliations:** ^1^ Department of Pediatric Endocrinology & Growth, Hirabai Cowasji Jehangir Medical Research Institute (HCJMRI), Pune, India; ^2^ Symbiosis School of Biological Sciences, Symbiosis International University, Pune, India; ^3^ Interdisciplinary School of Health Sciences, Savitribai Phule Pune University, Pune, India; ^4^ Department of Radiology, Jehangir Hospital, Pune, India; ^5^ Department of Radiology, KEM Hospital Research Centre, Pune, India

**Keywords:** metformin, carotid intima media thickness, dyslipidemia, insulin resistance, T1DM

## Abstract

**Introduction:**

Insulin resistance is being increasingly reported in type-1 Diabetes (T1D) and is known to accelerate microvascular complications. The Asian Indian population has a higher risk of double diabetes development compared to Caucasians. Hence, we studied the effect of adding Metformin to standard insulin therapy on glycemic control, insulin sensitivity (IS), cardiometabolic parameters and body composition in Indian adolescents with T1D.

**Methods:**

A Randomized controlled trial was conducted spanning 9 months (Registration number:CTRI/2019/11/022126). Inclusion: Age 10-19 years, T1D duration>1year, HbA1c>8% Exclusion: Uncontrolled vascular complications/comorbidities, Metformin intolerance, concomitant drugs affecting insulin sensitivity. Participants were randomized to Metformin/Placebo (n=41 each) groups and age, sex, duration-matched. Assessments were performed at baseline, 3 and 9 months.

**Results:**

82 participants aged 14.7 ± 3years (40 females) were enrolled, with a mean diabetes duration of 5.2 ± 2.3 years. Over 9 months, HbA1c decreased significantly by 0.8 (95% confidence interval: -1.2 to -0.3) from 9.8 ± 1.8% to 9.1 ± 1.7% on Metformin but remained largely unchanged (difference of 0.2, 95% confidence interval: -0.7 to 0.2) i.e. 9.9 ± 1.6% and 9.7 ± 2.2% on placebo. HbA1c improvement correlated negatively with baseline IS (EGDR:r= -0.3;SEARCH:r = -0.24, *p*<0.05) implying better HbA1c-lowering in those with decreased initial IS. CGM-based glycemic variability (standard deviation) reduced by 6.3 mg/dL (95% confidence interval: -12.9 to 0.2) from 100.2 ± 19.1 mg/dL to 93.7 ± 19.9 mg/dL in those on Metformin (p=0.05) but not placebo (94.0 ± 20.5; 90.0 ± 22.6 mg/dL). Insulin sensitivity: CACTIexa & SEARCH scores demonstrated no change with Metformin but significant worsening on placebo. Significant increase in LDL-C(42%), total cholesterol(133.6 to 151.1 mg/dL), triglyceride (60.0 to 88.0 mg/dL) and carotid intima-media thickness was noted on placebo but not Metformin. Weight, BMI, fat Z-scores increased significantly on placebo but not Metformin. Adverse events (AE) were minor; AE, compliance and safety parameters were similar between the two groups.

**Conclusion:**

Metformin as an adjunct to insulin in Asian Indian adolescents with T1D demonstrated beneficial effect on glycemic control, glycemic variability, IS, lipid profile, vascular function, weight and body fat, with a good safety profile when administered for 9 months.

## Introduction

The incidence of type-1 diabetes (T1D) among children and adolescents has increased worldwide (1). Insulin resistance (IR), a distinct feature of type-2 diabetes (T2D), is increasingly being evidenced in T1D (2). In patients with poor glycemic control, vascular dysfunction and its determinants (dyslipidemia, hypertension) are likely to develop earlier (3). This further deteriorates at adolescence; both, normal and obese adolescents with T1D have demonstrated higher IR when compared to body mass index (BMI)-matched healthy peers (4). IR accelerates micro and macrovascular complications in T1D. Aggressive glycemic control reduces but does not entirely eliminate the risk of development and progression of IR and complications in T1D (5–7).

A study by the authors’ group observed similar prevalence of obesity/metabolic syndrome in Indian youth with T1D compared to the non-diabetic population (8, 9). Adiposity predisposes to overproduction of insulin-antagonist hormones, free fatty acids, interference with insulin-mediated signal transduction and a pro-inflammatory state leading to increased insulin requirement further propagating adiposity, thus worsening IR in T1D (10).

Vascular dysfunction, a potentially reversible finding, develops much earlier than symptomatic cardiac disease; studies have demonstrated an increased carotid intima media thickness (cIMT, marker of early atherosclerosis) in children with T1D versus healthy peers, with an inverse association of estimated insulin sensitivity (IS) and cardiometabolic disease risk in adolescents with T1D (11–13).

Metformin, an oral antihyperglycemic drug approved for management of IR in T2D, suppresses hepatic glucose production, increases peripheral glucose utilization and possibly decreases intestinal glucose absorption (14, 15). Though global data have shown improvements in IS, adiposity, vascular dysfunction, and reduction in daily insulin doses in adolescents with T1D, studies on glycemic control and lipid profile have yielded mixed results and data on Indian adolescents are lacking (4, 11, 14, 16). The Asian Indian population is at a much higher risk of developing ‘double diabetes’ due to a higher tendency to develop insulin resistance in comparison with Caucasians. Moreover, the risk of metabolic syndrome has been reported at lower levels of adiposity and BMI in Indians compared to Caucasians (17, 18). Hence, it seems prudent to assess the efficacy of Metformin adjunct therapy in Indians with T1D in order to improve glycemic control and to prevent/reverse the development of cardiometabolic risk. To address this unmet need, we planned a randomized controlled trial with the following objectives: 1) to evaluate the effect of adding Metformin as an adjunct to standard insulin therapy for 9 months in Indian adolescents with T1D on glycemic control, insulin sensitivity and cardiometabolic parameters (primary objectives) 2) to assess the impact of Metformin on anthropometry and body composition parameters (secondary objectives).

## Materials and methodology

### Study design

A single center, parallel group, double-blinded, randomized, placebo-controlled 9-month trial (November 2019 to August 2020) was conducted in line with the principles of Declaration of Helsinki, following institutional ethics committee approval (dated 01/11/2019) and was registered with the Clinical Trials Registry - India (CTRI/2019/11/022126). Written informed consent was obtained from the parents and assent from adolescents below 18 years of age. Those above 18 years of age provided written informed consent. Though initially planned over 6 months, the duration had to be extended to 9 months owing to COVID-19 lockdown and the same was conveyed to the institutional ethics committee.

### Subjects

**Figure 1:**
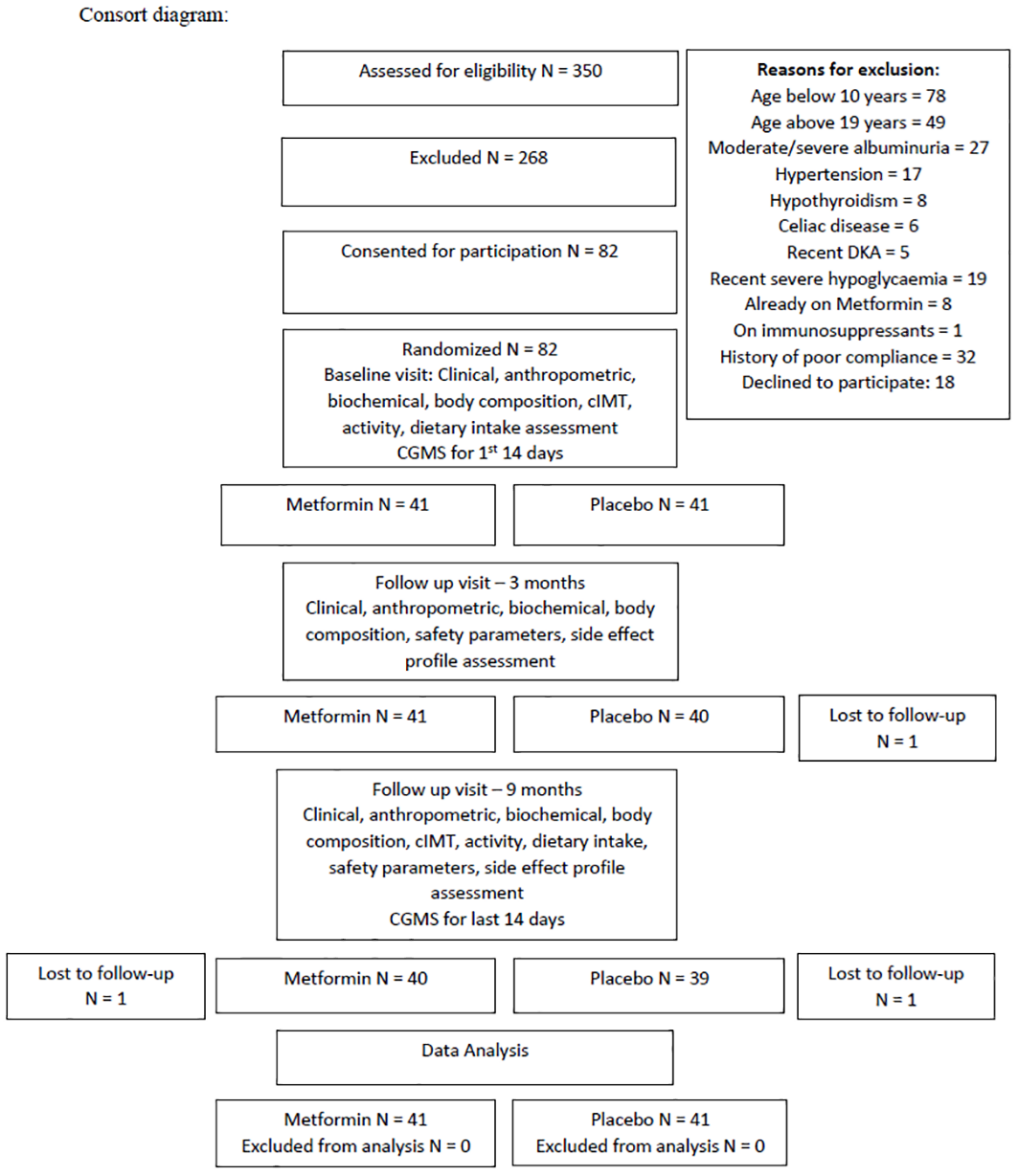
CONSORT diagram depicting the course of the randomized controlled trial.

All patients attending the Pediatric Type-1 Diabetes Clinic at our tertiary care center (Western Maharashtra, India) were screened for eligibility. Patients aged 10-19 years with T1D duration >1year, HbA1c >8% despite intensive insulin treatment were included. Those with uncontrolled complications/comorbidities (moderately or severely increased albuminuria, hypothyroidism, celiac disease, hypertension), on medications affecting IS (corticosteroids, immunosuppressants, statins), lack of treatment adherence/severe illness, history of diabetic ketoacidosis (DKA) within 60 days/severe hypoglycemic episode in the past 6 months prior to recruitment/>2 episodes of DKA in the previous year, or known hypersensitivity to Metformin were excluded (12). Thus, of 350 patients, 268 were excluded, 82 were willing and were included for participation (**Figure 1**). On performing an A-priori sample size calculation using G power 3.1.9.4, a total sample size of 80 was found to be sufficient to obtain a power of 0.8 with α=0.05, for repeated measure ANOVA between 2 groups with 3 time-points. Analysis was performed on an intention to treat basis.

### Methodology

The patients were randomized to the Metformin or Placebo groups (1:1 ratio) using sealed envelopes and were age, sex, diabetes duration matched, by the pharmacist. Both, participants and investigators, were blinded to the allocation. The dose of Metformin was determined based on the baseline body weight. Those weighing<60 kg received 500 mg Metformin twice daily, those weighing >60 kg received 1gm twice daily. Treatment was initiated at a dose of 500mg once daily and scaled up to the final dose over 2 weeks (12). Both tablets (Metformin and Placebo) were prepared by the same pharmacist and were identical in appearance and composition except for the active drug being tested i.e. Metformin hydrochloride (500 mg). During the intervention period, subjects were followed-up monthly to distribute tablets and were evaluated by a Pediatric Endocrinologist at each visit for T1D management, compliance and side-effects. At each visit, adolescents and their parents were counselled regarding dietary modifications based on their existing intake, by a single trained nutritionist. The importance of daily moderate to vigorous physical activity for 1 hour was emphasized. Weekly phone calls were conducted to assess safety and compliance parameters. As the RCT was double-blinded, the Pediatric Endocrinologist was not biased in terms of clinical management and both groups received identical dietary and lifestyle advice.

Anthropometric, biochemical, body composition parameters were measured at baseline, three and nine months from starting Metformin/placebo. Carotid intima media thickness (cIMT) was evaluated at baseline and endline. Safety parameters (Aspartate aminotransferase (AST), Alanine aminotransferase (ALT), lactate, number of hypoglycemic episodes and compliance were assessed at three and nine months. Due to poor feasibility of recalling patients every 15 days for sensor replacement, continuous glucose monitoring was performed for the first and last 14 days of the study for time in/above/below range of target glucose, estimated A1c (eA1c), coefficient of variation, standard deviation.

Questionnaires were used to obtain the following data: age, duration of diabetes, age at diabetes onset, insulin regimen and total daily dose of insulin. Activity was assessed using the Activity Questionnaire adapted for Indian children and adolescents (19, 20).

### Anthropometry

Height (Seca Portable stadiometer, up to 0.1 cm accuracy) and body weight (Seca 876 Flat scale, up to 100 g accuracy) were measured and BMI was computed (weight (kg)/height (m^2^)). World Health Organization (WHO) guide to physical measurements was used to measure waist (WC) and hip circumferences and waist:hip ratio (WHR) was calculated (21). Weight, height, WC and BMI were converted to Z-scores (22, 23).

### Body composition

Fat and fat-free mass were assessed using Bioelectrical Impedance Analyzer, (Tanita Model BC420MA) in standing position after at least 3 hours of fasting, and voiding before measurements and Z-scores were computed (24, 25).

### Biochemical parameters

6-8mL blood was drawn after a minimum of 8 hours fast; lipid profile [total cholesterol, triglycerides and high density lipoprotein cholesterol (HDL-C)] was measured using enzymatic method and low-density lipoprotein cholesterol (LDL-C) concentrations were calculated using Friedewald formula (26). Glycemic control was evaluated by measuring glycosylated hemoglobin (HbA1c) using high-performance liquid chromatography (HPLC, BIO-RAD, Germany). Serum Leptin and Adiponectin were measured by enzyme-linked immunoassay (Titerzyme EIA kit, Assay designs’ Inc, USA). AST, ALT were tested by International Federation of Clinical Chemistry (IFCC) method (fully automated analyser Selectra Pro S, Germany) and serum vitamin B12 was measured by chemiluminescence. Urinary spot albumin was assessed by immunoturbidimetry and urinary creatinine by Jaffe’s method.

### Carotid intima media thickness (cIMT)

cIMT was measured by a blinded, single radiologist using the ultrasound B mode. Far-wall cIMT was assessed from standard magnified images of the far (posterior) wall of the common carotid artery, immediately proximal to the carotid bulb. The maximum distance between the media-adventitia interface and the lumen-intima interface was recorded.

### Continuous glucose monitoring (CGM)

The Freestyle Libre Pro CGMS (Abbott, USA) was used for the first and last 14 days of the study period. All devices were fitted by the same trained personnel over the right upper arm. Participants and investigators were blinded to CGM glucose readings. After removal, data for each participant were downloaded. The time in, above and below range, estimated A1c (eA1c) averaged over the 14-day period, coefficient of variation, standard deviation were used for analysis (27).

### Insulin sensitivity indices

Though the gold standard for assessment of IS in T1D is by measuring the glucose disposal rate from a hyperinsulinemic-euglycemic clamp, it is cumbersome and invasive. Hence, we utilized calculated insulin sensitivity estimation equations as follows:

Estimated glucose disposal rate (EGDR in mg/kg/min) = 24.31 – 12.22(WHR) – 3.29(HTN) – 0.57(HbA1c,%) (28).

SEARCH = exp [4.64725 – 0.02032 (waist; cm) – 0.09779 (HbA1c; %) – 0.00235 (Triglyceride; mg/dl)] (29).

CACTI = exp (4.06154 - 0.01317 * waist [cm] - 1.09615 * insulin dose [daily units per kg] - 0.02027 * adiponectin [mcg/mL] - 0.27168 * triglycerides [mmol/L] - 0.00733 * DBP [mm Hg]) (13).

CACTI excluding adiponectin (CACTI exa) = exp (4.1075 - 0.01299 [waist, cm] - 1.05819 [insulin dose, daily units per kg] - 0.00354 [triglycerides, mg/dL] - 0.00802 [DBP, mm Hg]) (13).

### Statistical analysis

Data were analysed using SPSS 26.0 (IBM SPSS, Bangalore, India). All variables were tested for normality. Means (standard deviation) were used for normally distributed and medians (interquartile range) for non-normally distributed variables. The independent sample t-test was used for metformin versus placebo group comparisons at baseline, 3 months and 9 month’s time-frames for normally distributed variables and non-parametric tests for non-normally distributed variables. The changes in parameters from baseline (Metformin v/s Placebo) were compared between the two groups using the independent t-test. The general linear model for repeated measures (repeated measure ANOVA) was used to compare parameters at baseline, 3 and 9 months longitudinally in the metformin group and the placebo group for normally distributed variables, while the Friedman test was used for non-normally distributed variables. As change in fat mass Z-scores and waist circumference Z-scores demonstrated no correlation with HbA1c, total cholesterol, HDL-C, LDL-C, triglycerides, they were not adjusted for.

## Results

Of the 82 participants included in the study,1 was lost to follow-up at 2^nd^ visit, and 2 at 3^rd^ visit. The mean age of participants at baseline was 14.7 ± 2.9 years, 40 (49%) were female. Mean diabetes duration was 5.3 ± 2.2 and 5.1 ± 2.2 years among the treatment and placebo groups respectively. The mean HbA1c was 9.8 and 9.9% respectively. There were no statistically significant differences in the anthropometric, body composition, glycemic control, cardiometabolic, dietary, and physical activity parameters between the two groups at baseline (**Table 1**).

**Table 1:** Baseline characteristics of adolescents with type 1 Diabetes on Metformin versus Placebo.

		Baseline		
Baseline parameters		Metformin (41)	Placebo (41)	Total (82)
Age (years)		14.8 (3.1)	14.7 (2.8)	14.7 (2.9)
Gender	Girls	20 (49%)	20 (49%)	40 (49%)
	Boys	21 (51%)	21 (51%)	42 (51%)
Diabetes Duration (years)		5.3 (2.2)	5.1 (2.2)	5.2 (2.3)
Anthropometry:				
Height Z-score		-0.6 (1.0)	-0.4 (1.0)	- 0.5 (1.0)
Weight Z-score		-0.4 (0.8)	-0.5 (0.9)	- 0.5 (0.9)
BMI Z-score		-0.2 (0.8)	-0.4 (0.8)	- 0.3 (0.8)
Waist circumference (cm)		66.7 (8.5)	67.2 (9.4)	66.9 (8.6)
Waist Circumference Z-score		-1.7 (1.0)	-1.6 (1.1)	- 1.7 (1.1)
Hip circumference (cm)		81.5 (9.2)	81.9 (10.3)	81.7 (9.6)
Waist Hip Ratio		0.8 (0.1)	0.8 (0.1)	0.8 (0.1)
Body composition parameters:				
Fat percentage (%)		20.4 (9.8)	17.7 (10.1)	19.0 (10.0)
Fat Z-score		-0.2 (0.9)	-0.4 (0.9)	- 0.3 (0.9)
Muscle mass percentage (%)		32.7 (6.3)	33.3 (8.7)	32.9 (7.6)
Lean body mass Z-score		-3.0 (0.6)	-2.9 (0.7)	- 2.5 (0.9)
Glycemic control parameters:				
Time in range (%)		22.2 (12.4)	24.7 (13.1)	23.7 (12.4)
Time below range (%)		15.2 (10.1)	12.6 (9.6)	13.9 (9.8)
Time above range (%)		62.5 (18.7)	62.6 (20.5)	62.5 (18.9)
Coefficient of variation (%)		49.9 (11.5)	46.0 (10.0)	48.0 (10.1)
Standard deviation (mg/dL)		100.2 (19.1)	94.0 (20.5)	97.1 (20.2)
eA1c (%)		9.0 (2.3)	8.9 (1.8)	8.9 (2.0)
HbA1c (%)		9.8 (1.8)	9.9 (1.6)	9.9 (1.7)
Daily dose of insulin (Units/kg/day)		1.1 (0.3)	1.0 (0.3)	1.0 (0.3)
Cardiometabolic risk factors:				
Systolic blood pressure (mm Hg)		110.8 (6.3)	109.8 (9.4)	110.7 (8.0)
Diastolic blood pressure (mm Hg)		73.0 (5.8)	73.7 (7.8)	73.4 (6.8)
Leptin (mcg/mL)		8.0 (9.9)	5.3 (16.9)	4.8 (13.7)
Adiponectin (ng/mL)		18.3 (9.1)	18.1 (9.5)	18.2 (9.3)
Adiponectin/Leptin ratio		2.3 (9.4)	3.5 (8.2)	2.9 (9.3)
Average maximum cIMT (mm)		0.32 (0.05)	0.32 (0.05)	0.32 (0.05)
Total cholesterol (mg/dL)		141.9 (23.0)	133.6 (27.7)	137.7 (25.7)
Triglyceride (mg/dL)		61.7 (17.9)	60.0 (34.3)	56.5 (42.5)
HDL cholesterol (mg/dL)		51.7 (7.9)	51.3 (7.8)	51.5 (7.8)
LDL cholesterol (mg/dL)		77.8 (19.6)	67.2 (28.2)	72.5 (24.7)
Urinary albumin:creatinine ratio (mg/g)		9.4 (14.3)	12.1 (12.6)	10.8 (13.5)
Daily Physical Activity:				
Moderate activity (minutes/day)		47.3 (38.6)	55.0 (54.7)	51.1 (47.0)
Vigorous activity (minutes/day)		61.3 (45.4)	57.1 (51.5)	59.3 (48.2)
Insulin sensitivity indices:				
CACTI score		6.1 (4.0)	6.7 (3.5)	6.3 (3.6)
CACTIexa score		3.9 (1.3)	4.1 (1.8)	3.9 (1.6)
SEARCH score		9.2 (2.2)	9.0 (2.6)	9.1 (2.4)
EGDR score		8.6 (1.6)	8.4 (1.4)	8.5 (1.5)
Safety parameters:				
Vitamin B12 (pg/mL)		276.3 (144.8)	262.7 (123.0)	269.5 (133.6)
Lactate (mg/dL)		10.0 (3.3)	11.1 (3.6)	10.5 (3.5)
AST (IU/L)		15.9 (5.1)	15.9 (6.0)	15.9 (5.1)
ALT (IU/L)		14.8 (6.8)	17.1 (7.3)	15.9 (7.1)

There were no significant differences in any parameters between the Metformin and placebo groups at baseline (p > 0.1). Normally distributed data are described as Mean (SD), non-normally distributed data as Median (IQR) and ordinal data as value (%) [BMI, Body Mass Index; cIMT, Carotid intima media thickness; HDL, High density lipoprotein; LDL, Low density lipoprotein; VLDL, Very low density lipoprotein; AST, Aspartate aminotransferase; ALT, Alanine aminotransferase; CACTI, Coronary artery calcification in Type I diabetes; CACTIexa - Coronary artery calcification in Type I diabetes excluding adiponectin; SEARCH, Search for Diabetes in Youth study; EGDR, estimated glucose disposal rate].

### Efficacy analyses

#### Primary outcomes

##### Glycemic control

**Figure 2:**
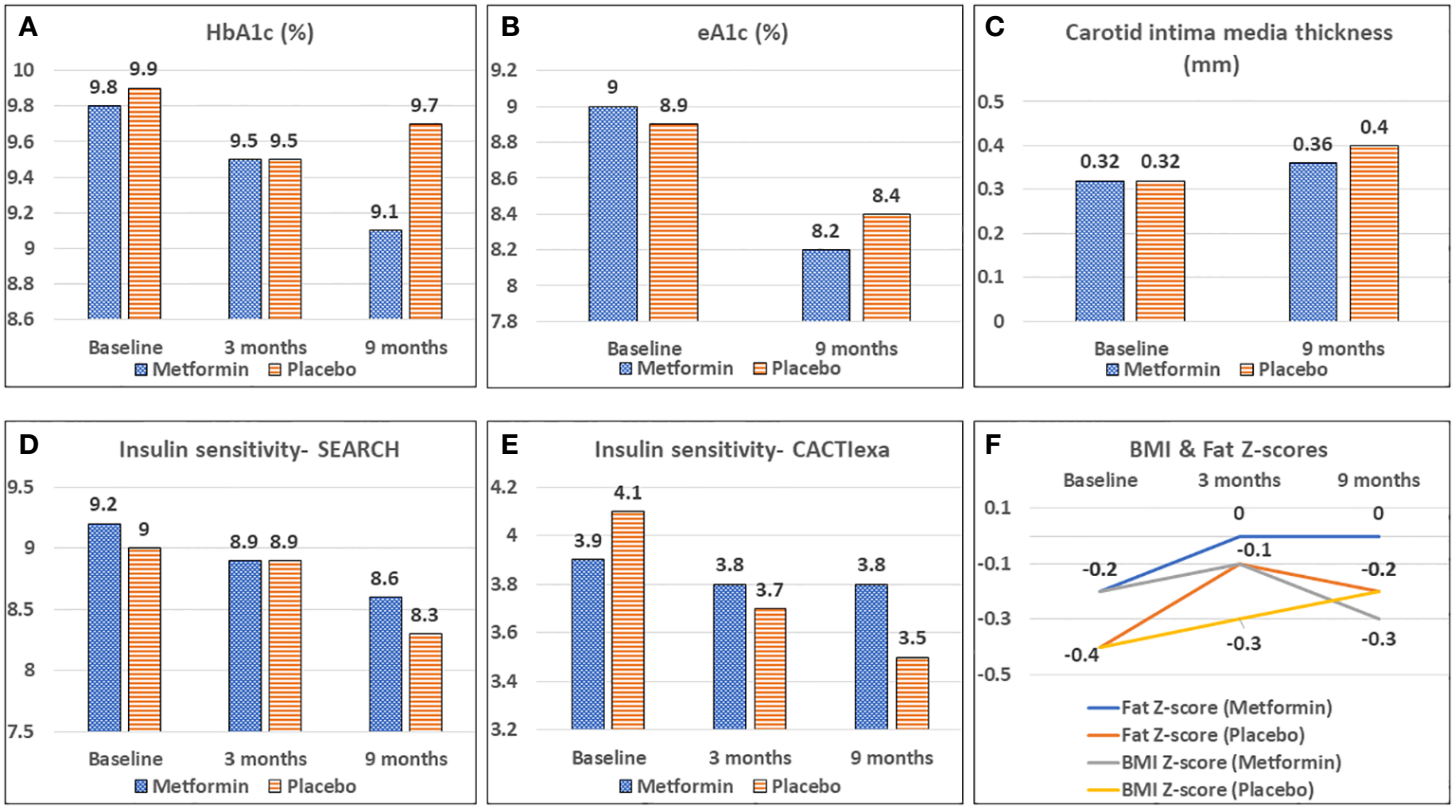
Effect of Metformin versus Placebo on glycemic control **(A, B)**, vascular health **(C)**, calculated insulin sensitivity **(D, E)** and body composition **(F)**.

During the study period, the mean HbA1c decreased significantly from 9.8% to 9.1% in the metformin group (*p*< 0.05) but remained unchanged (9.9 and 9.7%) in the placebo group over 9 months. (**Table 2**; **Figure 2A**) CGM parameters too showed an improvement in glycemic control as evidenced from a reduction in eA1c from 9.0 ± 2.3% to 8.2 ± 1.6% and in SD from 100.2 ± 19 mg/dL to 93.7± 19.9 mg/dL (*p*=0.05) in the Metformin group but not in the placebo group (**Figure 2B**). The improvement (difference) in HbA1c over 9 months correlated negatively with the baseline insulin sensitivity (EGDR: r= -0.3; SEARCH: r = -0.24, *p*< 0.05).

**Table 2:** Comparison of parameters at baseline, 3 months and 9 months.

Metformin group				Placebo group			
	Baseline	3 months	9 months		Baseline	3 months	9 months
Anthropometry:							
Height Z-score^A,C^	-0.6 (1.0)	-0.7 (1.0)	-0.7 (1.0)	Height Z-score	-0.4 (1.0)	-0.4 (1.0)	-0.4 (1.0)
Weight Z-score	-0.4 (0.8)	-0.3 (0.9)	-0.3 (0.9)	Weight Z-score ^A,C^	-0.5 (0.9)	-0.4 (0.9)	-0.4 (1.0)
BMI Z-score	-0.2 (0.8)	-0.1 (0.8)	-0.3 (0.9)	BMI Z-score^C^	-0.4 (0.8)	-0.3 (0.8)	-0.2 (0.9)
Waist circumference (cm) ^A,B,C^	66.7 (8.5)	68.6 (8.6)	70.8 (8.6)	Waist circumference (cm) ^C^	67.2 (9.4)	68.1 (9.7)	70.2 (10.5)
Waist circumference Z-score^C^	-1.7 (1.0)	-1.5 (1.0)	-1.1 (1.1)	Waist circumference Z-score	-1.6 (1.1)	-1.5 (1.1)	-1.3 (1.3)
Hip circumference (cm) ^B,C^	81.5 (9.2)	82.7 (9.5)	85.6 (10.3)	Hip circumference (cm) ^A,C^	81.9 (10.3)	83.5 (9.7)	84.6 (9.8)
Waist Hip Ratio	0.8 (0.1)	0.8 (0.1)	0.8 (0.1)	Waist Hip Ratio	0.8 (0.1)	0.8 (0.1)	0.8 (0.1)
Body composition parameters:							
Fat percentage (%)	20.4 (9.8)	22.0 (10.1)	22.4 (10.0)	Fat percentage (%) ^A,B,C^	17.7 (10.1)	19.9 (10.3)	20.3 (10.1)
Fat Z-score	-0.2 (0.9)	0.0 (0.9)	0.0 (0.9)	Fat Z-score ^A,C^	-0.4 (0.9)	-0.1 (0.9)	-0.2 (0.9)
Muscle mass percentage (%) ^B,C^	32.7 (6.3)	32.5 (7.3)	33.5 (6.9)	Muscle mass percentage (%) ^A,B,C^	33.3 (8.7)	34.2 (8.5)	35.4 (8.8)
Lean body mass Z-score	-3.0 (0.6)	-3.1 (0.7)	-3.0 (0.7)	Lean body mass Z-score	-2.9 (0.7)	-2.9 (0.6)	-2.9 (0.7)
Glycemic control parameters:							
Time in range (%)	22.2 (12.4)	–	26.0 (10.5)	Time in range (%)	24.7 (13.1)	–	26.9 (11.8)
Time below range (%)	15.2 (10.1)	–	14.1 (10.5)	Time below range (%)	12.6 (9.6)	–	13.7 (11.7)
Time above range (%)	62.5 (18.7)	–	59.9 (18.7)	Time above range (%)	62.6 (20.5)	–	59.3 (21.9)
Time in level 1 hypoglycemia (%)	4.8 (2.9)	–	5.0 (3.3)	Time in level 1 hypoglycemia (%)	4.0 (2.7)	–	4.9 (3.9)
Time in level 2 hypoglycemia (%)	7.3 (6.5)	–	8.8 (7.5)	Time in level 2 hypoglycemia (%)	6.6 (6.8)	–	8.8 (8.8)
Coefficient of variation (%)	49.9 (11.5)		50.6 (12.6)	Coefficient of variation (%)	46.0 (10.0)		48.5 (10.1)
Standard deviation (mg/dL)^C^	100.2 (19.1)		93.7 (19.9)	Standard deviation (mg/dL)	94.0 (20.5)		90.0 (22.6)
eA1c (%) ^C^	9.0 (2.3)	–	8.2 (1.6)	eA1c (%)	8.9 (1.8)	–	8.4 (2.2)
HbA1c (%) ^C^	9.8 (1.8)	9.5 (1.7)	9.1 (1.7)	HbA1c (%)	9.9 (1.6)	9.5 (1.6)	9.7 (2.2)
Daily dose of insulin (Units/kg/day)	1.1 (0.3)	1.0 (0.4)	1.0 (0.3)	Daily dose of insulin (Units/kg/day)	1.0 (0.3)	1.1 (0.3)	1.1 (0.3)
Cardiometabolic risk factors:							
Systolic BP (mm Hg)	110.8 (6.3)	110.2 (7.1)	110.9 (11.5)	Systolic BP (mm Hg)	109.8 (9.4)	111.4 (7.8)	111.9 (12.6)
Diastolic BP (mm Hg)	73.0 (5.8)	73.5 (5.6)	70.2 (9.1)	Diastolic BP (mm Hg) ^C^	73.7 (7.8)	73.2 (6.7)	69.4 (9.2)
Leptin (mcg/mL)	8.0 (9.9)	–	5.0 (3.7)	Leptin (mcg/mL)	5.3 (16.9)	–	5.5 (19.9)
Adiponectin (ng/mL)	18.3 (9.1)	–	15.1 (11.5)	Adiponectin (ng/mL)	18.1 (9.5)	–	16.3 (11.4)
A/L ratio	2.3 (9.4)	–	2.6 (9.6)	A/L ratio	3.5 (8.2)	–	3.2 (24.5)
Average maximum cIMT (mm)	0.32 (0.05)	–	0.36 (0.05)^D^	Average maximum cIMT (mm) ^C^	0.32 (0.05)	–	0.40 (0.05)^D^
Total cholesterol (mg/dL)	141.9 (23.0)	145.2 (27.9)	148.1 (31.5)	Total cholesterol (mg/dL) ^C^	133.6 (27.7)	136.4 (24.7)	151.1 (42.9)
Triglyceride (mg/dL)	61.7 (17.9)	64.0 (25.5)	68.0 (41.0)	Triglyceride (mg/dL) ^C^	60.0 (34.3)	88.0 (41.5)	88.0 (72.5)
HDL cholesterol (mg/dL) ^C^	51.7 (7.9)	50.5 (8.3)^D^	46.8 (7.8)	HDL cholesterol (mg/dL) ^A^	51.3 (7.8)	47.3 (5.3)^D^	48.5 (7.5)
LDL cholesterol (mg/dL)	77.8 (19.6)	80.1 (25.9)	85.7 (30.9)	LDL cholesterol (mg/dL) ^C^	67.2 (28.2)	72.4 (25.2)	83.3 (41.0)
Urinary albumin:creatinine ratio (mg/g)	9.4 (14.3)	–	15.7 (17.9)	Urinary albumin:creatinine ratio (mg/g)	12.1 (12.6)	–	17.5 (21.5)
Daily Physical Activity:							
Moderate activity (minutes/day)	47.3 (38.6)	–	47.5 (38.5)	Moderate activity (minutes/day)	55.0 (54.7)	–	54.5 (55.7)
Vigorous activity (minutes/day) ^C^	61.3 (45.4)	–	40.0 (35.0)	Vigorous activity (minutes/day) ^C^	57.1 (51.5)	–	39.5 (32.3)
Dietary intake:							
Proteins (gm/kg/day)	0.8 (0.4)	–	0.9 (0.3)	Proteins (gm/kg/day)	0.8 (0.4)	–	0.9 (0.4)
Fat (gm/kg/day) ^C^	0.9 (0.4)	–	1.2 (0.6)	Fat (gm/kg/day) ^C^	0.9 (0.4)	–	1.1 (0.5)
Carbohydrates (gm/kg/day)	4.6 (1.7)	–	5.1 (1.5)	Carbohydrates (gm/kg/day)	4.7 (1.7)	–	5.2 (1.9)
Fibre (gm/kg/day)	0.5 (0.2)	–	0.5 (0.1)	Fibre (gm/kg/day) ^C^	0.5 (0.2)	–	0.6 (0.2)
Insulin sensitivity indices:							
CACTI	6.1 (4.0)	–	6.2 (4.7)	CACTI	6.7 (3.5)	–	5.7 (3.9)
CACTIexa	3.9 (1.3)	3.8 (1.5)	3.8 (1.9)	CACTIexa^A,C^	4.1 (1.8)	3.7 (1.5)	3.5 (1.7)
SEARCH	9.2 (2.2)	8.9 (2.1)	8.6 (2.4)	SEARCH^C^	9.0 (2.6)	8.9 (2.6)	8.3 (2.7)
EGDR	8.6 (1.6)	8.8 (1.2)	8.7 (1.4)	EGDR	8.4 (1.4)	8.7 (1.6)	8.5 (1.8)
Safety parameters:							
Vitamin B12 (pg/mL)	276.3 (144.8)	–	248.8 (98.5)	Vitamin B12 (pg/mL)	262.7 (123.0)	–	260.9 (121.7)
Lactate (mg/dL)^A,C^	10.0 (3.3)	11.8 (4.8)	12.2 (5.9)	Lactate (mg/dL)	11.1 (3.6)	–	12.7 (7.4)
AST (IU/L)^A^	15.9 (5.1)	18.8 (7.2)	16.2 (4.0)	SGOT (IU/L)	15.9 (6.0)	18.2 (8.3)	17.4 (4.4)
ALT (IU/L)	14.8 (6.8)	16.9 (6.1)	14.5 (3.8)	SGPT (IU/L)	17.1 (7.3)	16.7 (8.6)	16.9 (6.4)

Normally distributed data are mentioned as Mean (SD) and non-normally distributed data as Median (IQR).

^A^ indicates significant difference between parameters at baseline and 3 months (p< 0.05).

^B^ indicates significant difference between parameters at 3 months and 9 months (p< 0.05).

^C^ indicates significant difference between parameters at baseline and 9 months (p< 0.05).

^D^ indicates difference between the Metformin and Placebo groups (p< 0.05).

BMI, Body Mass Index; cIMT, Carotid intima media thickness; HDL, High density lipoprotein; LDL, Low density lipoprotein; VLDL, Very low density lipoprotein; AST, Aspartate aminotransferase; ALT, Alanine aminotransferase; CACTI, Coronary artery calcification in Type I diabetes; CACTIexa - Coronary artery calcification in Type I diabetes excluding adiponectin; SEARCH, Search for Diabetes in Youth study; EGDR, estimated glucose disposal rate.

##### Daily Insulin dosage

There was no change in the insulin requirement of either group at 3 or 9 months of intervention.

##### Insulin sensitivity

**Figure 3:**
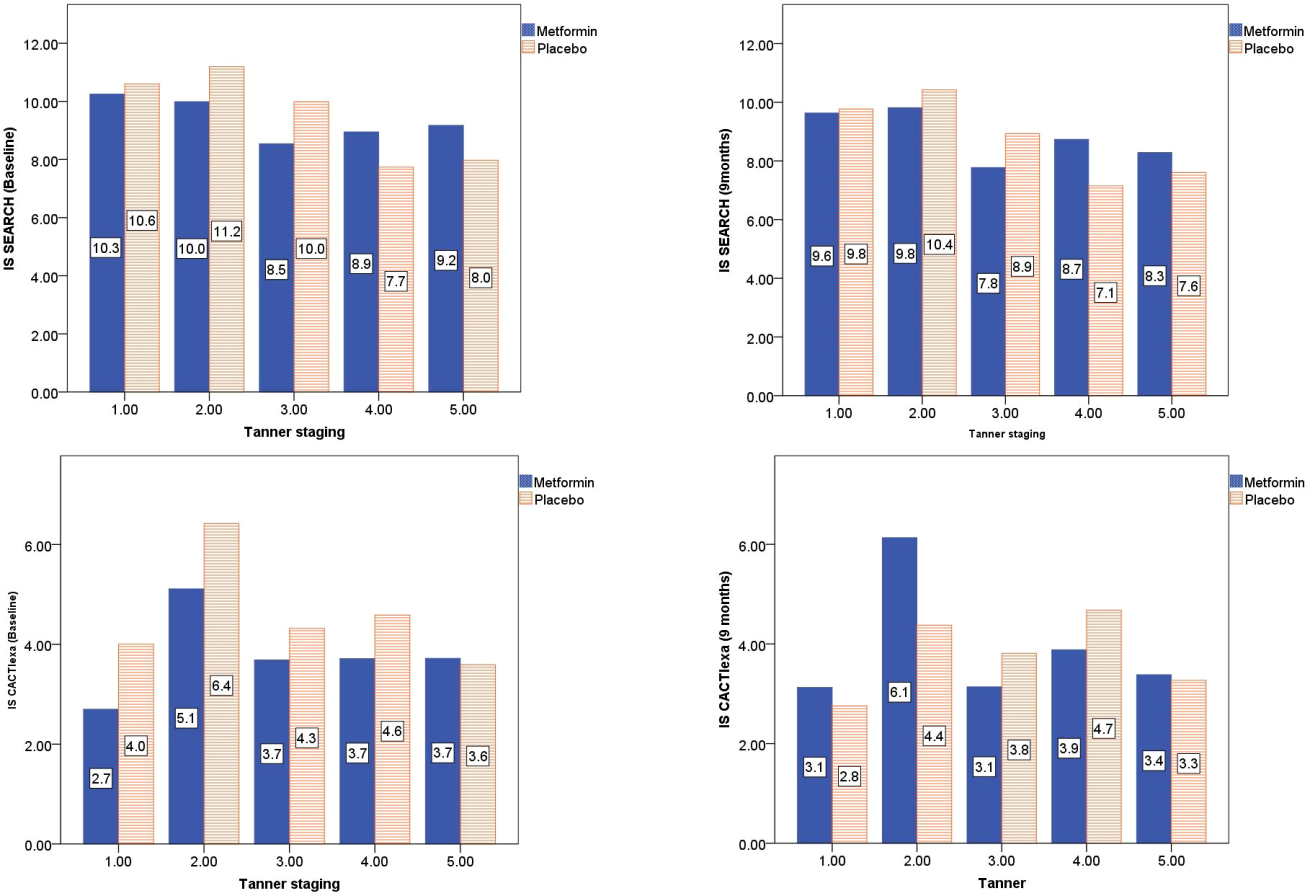
Tanner stage-wise stratification of calculated insulin sensitivity at baseline and 9 months of Metformin versus Placebo use.

The CACTIexa and SEARCH scores revealed no worsening of IS among those on Metformin, but a significant worsening over 9 months was observed among those on placebo (*p<*0.05, **Table 2**; **Figures 2D, E**). **Figure 3** depicts insulin sensitivity at baseline and endline stratified as per Tanner sexual maturity staging.

##### Cardiometabolic factors

**Figure 4:**
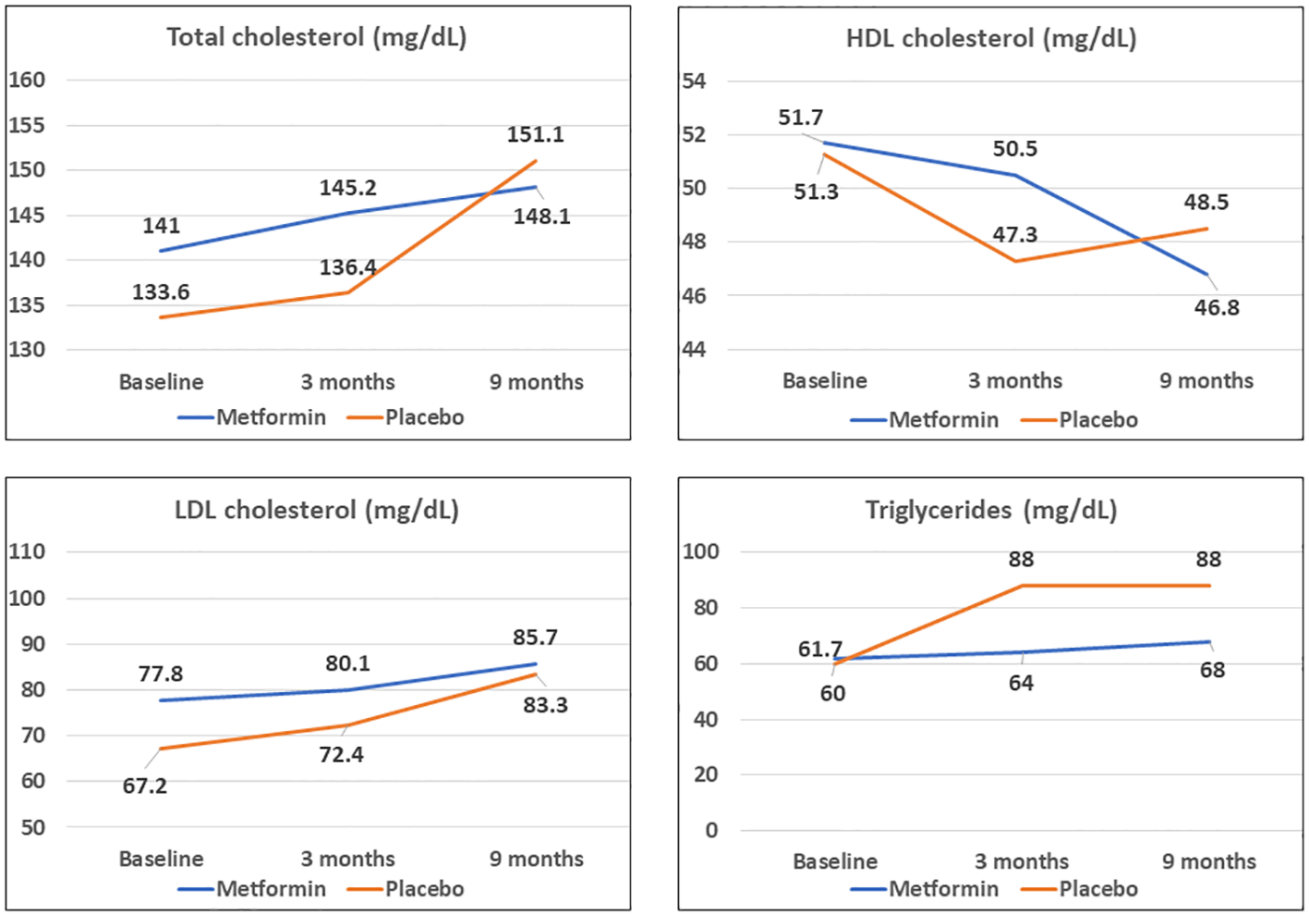
Comparison of trends in lipid profile parameters among Metformin and Placebo groups at baseline, 3- and 9-months duration.

**Figure 5:**
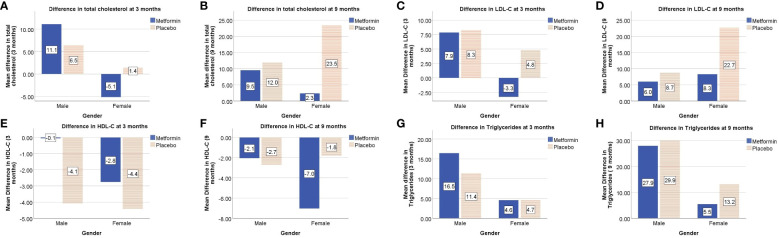
Gender-wise differences in lipid profile parameters from baseline i.e total cholesterol at 3 and 9 months (**A**, **B** respectively), LDL-C at 3 and 9 months (**C**, **D** respectively), HDL-C at 3 and 9 months (**E**, **F** respectively), triglycerides at 3 and 9 months (**G**, **H** respectively).

At 3 months, the HDL-C concentrations were significantly lower in the placebo group as compared to the Metformin group (*p<* 0.05). However, this beneficial effect of Metformin on HDL-C was not observed at 9 months (**Table 2**). A significant (42%) increase in the LDL-C concentrations from baseline was observed in the placebo group as compared to the Metformin group (14%) at 9 months (*p*=0.04). The placebo group demonstrated a significant increase in the total cholesterol (from 133.6 to 151.1 mg/dL, *p*< 0.05) and triglycerides (60.0 to 88.0 mg/dL) over 9 months, which was not observed among those on Metformin. There was significant worsening of all lipid profile parameters except HDL-C in the placebo group, which was not observed in the Metformin group (**Figures 4**, **5**). The average maximum cIMT at 9 months was significantly higher (worse) in the placebo group compared to the Metformin group (*p*< 0.05, **Figure 2C**). However, this difference was not observed after adjusting for change in HbA1c.

#### Secondary outcomes

##### Anthropometry and body composition

A significant increase in the weight Z-score was observed in the placebo group over 9 months, but not in the Metformin group. Similarly, the BMI Z-scores and Fat Z-scores worsened among the placebo group, but not the Metformin group (**Figure 2F**). However, waist circumference Z-scores increased significantly over 9 months among those on Metformin versus those on placebo (**Table 2**).

##### Diet and activity

There was a significant reduction in vigorous activity among both the groups over 9 months, coinciding with the COVID lockdown. Similarly, a significant increase in the dietary fat content per kg body weight was noted among both groups, and a significant increased fibre intake was observed in the placebo group (**Table 2**).

##### Safety data

A significant increase in the lactate concentration was observed following Metformin treatment for 9 months (**Table 2**). However, both baseline and endline values were within the reference range.

#### Compliance and side effects 

Overall compliance was similar across the two groups (Metformin: 92.5 ± 8.1%, Placebo: 93.6 ± 10.1%; *p* = 0.6). Compliance lower than 70% was observed only in 4 patients (2 from each group). The 2 patients from the metformin group attributed it to nausea and vomiting, while one patient each from the placebo group attributed it to vomiting and abdominal pain respectively. Among those on Metformin, 4 developed nausea/vomiting and 1 developed diarrhoea during the initial 3 months of intervention, while one each from the placebo group developed vomiting and abdominal pain (**Table 3**). Between 3 to 9 months, two patients from the Metformin group complained of nausea/vomiting and 1 complained of abdominal pain. The dose of Metformin was temporarily halved for a week among those experiencing gastrointestinal disturbances. There was no significant increase in the time spent in level 1 (glucose levels between 54-70 mg/dL) and level 2 (glucose levels< 54mg/dL) hypoglycemia among those on Metformin compared to placebo. None of the participants experienced severe or persistent hypoglycemia and hence dose reduction was not warranted. Isolated episodes of hypoglycemia detected by self-monitored blood glucose assessment were treated with oral glucose (0.3 g/kg body weight). There were no differences in the proportion of participants developing more than 5 hypoglycemic episodes per month (self-monitored) at any time point in the study.

**Table 3:** Comparison of adverse effect profiles between Metformin and Placebo groups.

Adverse effects:	Metformin (N=41)	Placebo (N=41)	Total (N=82)	P value
At baseline				
5 or more hypoglycemic episodes per month	2 (4.8%)	4 (10.3%)	6 (7.6%)	0.34
At 3 months				
Nausea/vomiting	4 (9.8%)	1 (2.5%)	5 (6.2%)	0.17
Diarrhoea	1 (2.5%)	0 (0.0%)	1 (1.2%)	0.31
Abdominal pain	0 (0.0%)	1 (2.5%)	1 (1.2%)	0.31
5 or more hypoglycemic episodes per month	13 (31.6%)	9 (22.5%)	22 (26.9%)	0.35
At 9 months				
Nausea/vomiting	2 (5.0%)	0 (0.0%)	2 (2.5%)	0.15
Diarrhoea	0 (0.0%)	0 (0.0%)	0 (0.0%)	–
Abdominal pain	1 (2.5%)	0 (0.0%)	1 (1.2%)	0.31
5 or more hypoglycemic episodes per month	10 (25%)	5 (12.5%)	15 (18.2%)	0.15

## Discussion

The current study demonstrates the safety and efficacy of Metformin adjunct therapy in T1D. We report a significant favourable effect of Metformin on glycemic control (HbA1c), glycemic variability (SD), insulin sensitivity, body weight and body composition (adiposity) as well as a cardiometabolic protective effect in Indian adolescents with type-1 diabetes, despite an increased dietary fat intake and reduced physical activity during lockdown. Metformin was found to be safe, with minor side effects (without significant differences compared to placebo).

We report an improvement in glycemic control on Metformin therapy; this finding is in consonance with other studies. A study by Sarnblad et al. on adolescents with poorly controlled T1D demonstrated a significant improvement in Hba1c (9.6 to 8.7%) and IS among those receiving Metformin for 3 months, without any effect on insulin requirement, body weight, waist and hip circumferences or blood lipid concentrations (14). Bjornstad et al. observed improvement in IS but not in HbA1c; this difference remained significant even after adjusting for change in BMI. Reduction in weight, BMI, fat mass, daily insulin dose/kg was observed, without any changes in traditional cardiovascular risk factors (blood pressure, lipid profile, HbA1c) following Metformin adjunct use (4). In a 12-month RCT involving 8–18-year-old Australian participants, Anderson et al. observed a favourable effect of Metformin on HbA1c at 3 months, but not subsequently, with no significant effects on traditional cardiometabolic parameters (BMI, waist circumference, adiponectin/leptin ratio, body fat percentage, lipids). A reduction in total daily insulin dose by 0.2 U/kg/day and an improvement in calculated IS was observed following 12 months of Metformin (11). In a meta-analysis of RCTs on the effect of Metformin in adolescents with T1D, Liu et al. reported a slightly lower HbA1c level in the Metformin group, with subgroup analysis revealing significant improvement in the general group but no significant changes in overweight/obese participants (15).

A trend towards reduction in glycemic variability (CGM-based SD) was noted following Metformin use. However, results were not consistent as this effect was not observed on the other CGM metric of variability i.e. CV. Recent evidence points towards the association of diabetic microvascular complications with glycemic excursions (30). Metformin may play a role in reducing glycemic variability thereby reducing the risk of vascular complications, though further studies are needed to consolidate this finding.

One of the proposed mechanisms for improvement in glycemic control in T1D following Metformin use is by an increase in insulin sensitivity; this finding is supported by our observation that the IS worsened among participants on placebo. We observed that the change in HbA1c correlated negatively with the baseline IS. Sarnblad et al. too have observed similar findings (14). This possibly suggests that the effect of Metformin on improving peripheral IS could be the plausible mechanism for the reduction in HbA1c in T1D, although simultaneous effects on hepatic glucose production cannot be excluded (14). The impact of metformin on IS in our study was not limited to overweight adolescents with T1D (as most of the participants had normal weight and BMI Z-scores).

Although improvement in IS should translate into a reduction in insulin requirement, this was not observed in our study. The possible explanation for this may be that our patients were selected on the basis of intermediate/poor glycemic control despite weight and pubertal stage-based insulin dosages, and not based on higher insulin requirement. Thus, a reduction in insulin dosage was not the main goal; whether Metformin given for a longer duration would reduce the insulin requirement in our cohort remains debatable. Both, Nwosu and Cree-Green et al. have reported no reductions in daily insulin requirement/kg despite improvement in glycemic control, BMI Z-scores and IS in obese adolescents with T1D (31, 32).

Traditionally, weight, BMI, waist, hip circumferences, dyslipidemia have been monitored as risk factors for cardiometabolic disease. These act either by worsening IR, and/or developing a pro-atherogenic milieu. However, both, IR and atherogenic environment have been demonstrated even in non-obese T1D (4, 33). Hence, newer methods of assessing cardiometabolic risk include estimation of vascular structural (cIMT) and functional (endothelial, smooth muscle) changes, adiponectin/leptin ratio. Though established cardiovascular disease in T1D manifests in adulthood, early vascular dysfunction is evident even in adolescence; IR is known to accelerate these changes (4, 34). We observed a beneficial effect of Metformin on cIMT over 9 months, however, significance was lost following adjustment for change in HbA1c suggesting improved glycemic control as the underlying mechanism (35, 36). Bjornstad et al. observed a reduction in phase-contrast MRI-derived maximal aortic wall shear stress in the ascending aorta as well as in aortic stiffness among those on Metformin. They observed that following Metformin use, the far wall cIMT improved significantly even after adjusting for changes in BMI/systolic blood pressure, but lost significance after adjusting for change in glucose infusion rate/insulin/kg (4). In contrast, an RCT found no significant effect of metformin on mean cIMT but observed improvement in glyceryl trinitrate–mediated dilatation of the brachial artery independent of HbA1c following 12 months of Metformin in 8-18 year-old patients with T1D, but did not observe any changes in cIMT, aortic IMT, BMI, blood pressure, lipids or other traditional cardiometabolic risk factors (11). A potential explanation for the differences in cIMT findings in this latter study was the inclusion of younger and prepubertal youth who are less likely to have derangements in cIMT.

Metformin use demonstrated a beneficial effect on total and LDL cholesterol and triglyceride concentrations in our study. Few other studies have reported these findings. Lund et al. reported significant lowering of total and LDL cholesterol in adults with T1D on Metformin adjunct therapy, despite no changes in HbA1c (16). In our study, the beneficial effect of Metformin on HDL-C was observed only at 3 months, but not thereafter. A possible explanation for this could be a significant increase in fat intake among both the groups during the lockdown. Metformin has been shown to improve the function rather than the concentration of HDL-C and may have led to similar effects in our study (37).

The current study observed lesser metabolic derangements over time in the Metformin group. Both, insulin sensitivity and lipid parameters worsened among those on placebo. Improvement in metabolic parameters following Metformin use occurs by multiple mechanisms, the most important being improvement in insulin sensitivity (10). Metformin also has additional direct effects on lipid concentrations by increasing the activity of lipoprotein lipase, thereby lowering triglycerides, total and LDL cholesterol (10).

Presence of obesity/adiposity worsens IR and accelerates vascular complications in T1D. Studies have shown that Metformin induces weight loss along with improvement in IS (4, 38). In our study, Metformin prevented significant increase in weight, BMI and fat Z-scores despite a significant increase in diet and reduced activity. This finding is relevant in the Asian Indian scenario as insulin resistance has been demonstrated in Indians at a much lower BMI as compared to Caucasians (17, 18). Thus, prevention of increased adiposity in T1D following Metformin use could play a role in the prevention of double diabetes. A pilot study by the authors’ group demonstrated the efficacy of Metformin in the prevention of double diabetes in Indian adolescents wherein the odds ratio and relative risk of developing double diabetes were 2.0 and 1.4 respectively in the placebo group, in comparison with those on Metformin (39). A systematic review and meta-analysis on the effect of Metformin on adolescents with T1D reported a significant reduction in BMI and body weight, with subgroup analysis depicting a significant reduction in overweight/obese participants and a trend in general participants (15).

A major unanticipated event that occurred during the study period was the COVID lockdown which resulted in decreased physical activity and binge eating, both these factors being known to influence IS negatively (40). Another study conducted by the authors’ group (Shah et al) during similar time period evaluated the impact of lockdown restrictions on children and youth with T1D and observed a significant decrease in physical activity as well as increase in waist circumference, body fat percentage and deterioration of lipid parameters and IS (41). Considering the same conditions prevailed for the current study cohort, decreased activity, increased dietary intake and waist circumference were observed among both the groups which could be attributed to the lockdown. However, despite these conditions, patients on Metformin did not develop significant weight gain, increase in BMI, fat percentage, total or LDL cholesterol or triglycerides in the current study. In comparison to Shah et al’s results which showed an overall improvement in HbA1c during the lockdown, participants from the current study showed an improvement only in the Metformin group but the mean HbA1c of participants on placebo remained unchanged (41). This could possibly be explained by differences in inclusion criteria of both the studies (current study included adolescents with poor glycemic control, the study by Shah et al. included all patients with T1D between 2-21 years of age) (41).

The current study found Metformin to be a safe drug with few minor side effects. There was no increase in the frequency or severity of hypoglycemic episodes on Metformin therapy. Similar to our study, most others have not reported any significant increase in symptomatic or severe hypoglycemic episodes (11). Thus, Metformin is not only an efficacious drug but also safe in adolescents with T1D. The commonest side effects reported with Metformin are gastrointestinal disturbances like nausea, vomiting and diarrhoea, most of which are mild and self-limiting (11, 15). Though Anderson et al. noted a significant reduction in vitamin B12 levels following Metformin use for 12 months, post-treatment values were within reference range (11).

To conclude, Metformin adjunct therapy in Asian Indian adolescents with T1D demonstrated a favourable effect on glycemic control, glycemic variability, insulin sensitivity, lipid profile, vascular function, body mass index and body fat composition with a good safety profile when administered for 9 months.

## Strengths and limitations

CGM was performed only for first and last 14 days of the study and may not be representative of the overall glycemic control during the study. However, the trends in HbA1c and CGM (eA1c, SD) were similar, showing a definite improvement in glycemic control after Metformin therapy. Small dense LDL-C is more closely associated with cardiovascular risk than total LDL-C, however, it could not be assessed in this study. Another limitation of our study is that, since patient-reported hypoglycemia was assessed, subclinical/asymptomatic hypoglycemia might have been overlooked. However, CGM metrics did not demonstrate increase in hypoglycemia. Though most other similar studies have been conducted over 6 months (compared to the current study spanning 9 months), further long-term studies are required to consolidate cardiometabolic outcome results (15, 35). Finally, as we excluded patients with diabetic complications like hypertension, albuminuria, the effect of Metformin on these conditions in our cohort remains to be assessed.

Excellent participant adherence with respect to medication and visits was our major strength. The study cohort included patients belonging to lower-middle and lower socioeconomic strata who receive free insulin and consumables from our out-patient clinic, making it one of the only studies to study effect of Metformin adjunct therapy in this economic stratum. Secondly, baseline weight/BMI were not criteria for patient selection; rather, our patients were matched (automatically) for baseline BMI and HbA1c thus helping to delineate the effects of Metformin better. Various other studies have evaluated the effect of Metformin exclusively in obese adolescents with T1D. In contrast, despite the proportion of overweight/obese adolescents in our study being minimal, and majority weighing normal for age and sex, the beneficial effects of Metformin adjunct therapy were evident. Thus, our results may be applicable to patients of a wider weight range, which is particularly relevant in the Asian Indian scenario, given the high risk of metabolic syndrome despite the absence of gross adiposity. Further global multicentre studies are needed to consolidate these findings in non-obese individuals with T1D, as differences in insulin resistance exist not only across different spectra of weight and adiposity, but also across races/ethnicities (10).

## Data availability statement

Data will be shared on reasonable request to Dr. Anuradha Khadilkar (anuradhavkhadilkar@gmail.com).

## Ethics statement

This study involving humans was approved by Ethics Commitee Jehangir Clinical Development Center Pvt. Ltd. The study was conducted in accordance with the local legislation and institutional requirements. Written informed consent for participation in this study was provided by the participants’ legal guardians/next of kin.

## Author contributions

SM: Conceptualization, Formal analysis, Methodology, Writing – original draft, Writing – review & editing. SK: Methodology, Writing – review & editing. NS: Formal analysis, Writing – review & editing. AK: Conceptualization, Formal analysis, Methodology, Supervision, Validation, Writing – original draft, Writing – review & editing. CO: Formal analysis, Methodology, Writing – review & editing. SB: Methodology, Writing – review & editing. KG: Formal analysis, Writing – review & editing. AW: Writing – review & editing. NK: Formal analysis, Supervision, Writing – review & editing. VK: Conceptualization, Formal analysis, Supervision, Writing – review & editing.
